# Nothing Iffy about HIF in the Hypothalamus

**DOI:** 10.1371/journal.pbio.1001116

**Published:** 2011-07-26

**Authors:** Sam Virtue, Antonio Vidal-Puig

**Affiliations:** University of Cambridge Metabolic Research Laboratories, Institute of Metabolic Science, Addenbrooke's Hospital, Cambridge, United Kingdom

## Abstract

Two crucial biological processes are (1) the sensing and coordination of responses to low oxygen levels and (2) the control of food intake and energy expenditure. The hypoxia-inducible factor (HIF) family of proteins is known to regulate responses to low oxygen, whereas neuropeptides derived from proopiomelanocortin (POMC) are implicated in the control of food intake and energy expenditure. It is now becoming apparent that these two apparently disparate processes may be linked, with the exciting discovery that HIF proteins can act in the brain to regulate food intake and energy expenditure as reported in the current issue of *PLoS Biology*. This primer discusses the traditional role of HIF proteins in terms of responding to oxygen levels in the periphery and also their new role in coordinating responses to nutrients in the brain through regulation of POMC.

## The Importance of Sensing Oxygen

Oxygen is one of the most critical molecules for life on earth, allowing far more energy to be extracted from nutrients than can be obtained from anaerobic metabolism. In light of their critical requirement for oxygen, aerobic organisms have developed sophisticated mechanisms to both sense oxygen and adapt to temporary or even prolonged reductions in oxygen levels.

**Figure 1:**
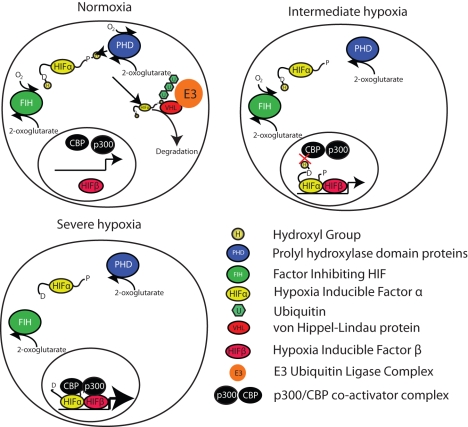
Mechanism of HIF activity. Under normoxic conditions, HIFα subunits are hydroxylated on proline residues. Hydroxylated prolines are recognised by the von Hippel-Lindau protein, ubiquinated by the E3 ubiquitin ligase, and targeted for proteosomal degradation. As oxygen levels fall, HIFα is stabilised and enters the nucleus to form a transcriptional complex with HIFβ subunits. FIH activity is maintained at lower oxygen levels than PHDs and remains active, hydroxylating asparagines. Hydroxylation of asparagines by FIH prevents association of the CBP/p300 coactivator complex with the HIFα/HIFβ transcriptional dimer. Under very low oxygen conditions, FIH becomes inactive and maximal HIF transcriptional activity is promoted.

The major site of oxygen utilisation in aerobic organisms is in mitochondria. However, mitochondria respire effectively down to oxygen levels as low as 1%. By the time oxygen levels have fallen this far, there is precious little opportunity left to adapt to reduced oxygen availability, and the organism is on course for a rapid and untimely death. In order to prevent precipitously low levels of oxygen being reached, organisms have evolved systems to sense and respond to oxygen levels up to 10%—a level of oxygenation much higher than almost any cell in the body would be exposed to [1]–[3]. Therefore, even under normoxic (normal oxygen levels) conditions, systems exist that can sensitively detect even small fluctuations in oxygen levels (Figure 1).

## How Is Oxygen Sensed?

In order to prevent a sudden loss of mitochondrial oxygen availability a complex system centred on the HIF1 family of transcription factors has evolved. HIF1α, HIF2α, and HIF3α represent three transcription factors, which are degraded under conditions of low oxygen [4],[5]. HIF proteins do not actually sense oxygen, that role is played by two classes of protein, proline hydroxylase domain (PHD)–containing proteins and factor inhibiting HIF (FIH) [6]. Both PHD and FIH proteins post-translationally modify HIF by hydroxylating amino acid residues. PHDs hydroxylate prolines located on HIFα subunits, rendering them targets for proteosomal degradation.

When oxygen levels fall and the activity of PHDs drop, HIFα subunits are no longer targeted for degradation. HIF1α and HIF2α subunits translocate to the nucleus and form a transcriptional complex with HIFβ. The role of HIF3α is less clearly defined and it can act to suppress HIF1α function [7]. HIF transcriptional complexes subsequently drive the expression of a wide variety of pathways all aimed at maintaining precious intracellular oxygen levels. FIH also post-translationally modifies HIFα in response to low oxygen, but does so by hydroxylating asparagines. Instead of targeting HIF for degradation, FIH alters the ability of HIF to interact with transcriptional coactivators such as CBP/p300 [8],[9]. FIH is more sensitive to oxygen than PHDs, thus even when PHDs are completely inactive and HIF fully stabilised, FIH can continue to regulate the degree of HIF activation. That said, it is likely that FIH and PHDs are active to a certain degree across a range of oxygen levels. Therefore, these two separate oxygen-sensing proteins allow the coordinated regulation of both the stability of HIFα subunits and, when stabilised, their degree of transcriptional activity.

## What Intracellular Metabolic Processes Are Targeted by HIF?

HIF activation is capable of regulating almost all aspects of cellular metabolism, with its central aim being to switch metabolism toward processes that require less oxygen. To this end, activation of HIF switches cells away from oxygen-intensive mitochondrial oxidative phosphorylation of glucose and beta-oxidation of lipids and promotes anaerobic glycolysis as a source for ATP [10],[11].

## Classical Roles for HIF

In addition to studies on the effects of experimentally manipulated oxygenation levels, the role of HIF has principally been studied in states where oxygenation levels are central to the pathogenicity of a given disease state. First, considerable interest has focused on the role of HIF in cancer growth. Cancers grow in an uncontrolled manner, with large solid tumour masses becoming profoundly hypoxic. Understanding how hypoxia is regulated within tumours may allow the design of new treatments to prevent tumour growth [12]. Equally, the role of HIF proteins in ischaemic diseases such as myocardial infarction and stroke has been extensively studied and characterised. Finally, HIF proteins were actually identified owing to their role in regulating the blood-boosting hormone erythropoietin. Prolonged exposure to altitude leads to an activation of HIF, which in turn increases transcription of erythropoietin and thus promotes the production of red blood cells. Drugs targeting the PH-HIF axis are under investigation as potential treatments for anaemia [13],[14]. More recently the role of HIF proteins in metabolic disease has come into focus. Trayhurn and colleagues first suggested that obesity is a disease state in which localised hypoxia within adipose tissue may mediate many of the metabolic alterations that occur within this tissue [15].

## Control of Whole Organism Energy Homeostasis by the Hypothalamus

Virtually all metabolic processes have some component of central (brain-mediated) control. Many of these processes are controlled by regulatory circuits located in the hypothalamus. For example, one of the most important neuronal populations for the regulation of food intake is the POMC-expressing neurons, particularly those within the hypothalamus, which produce the neuropeptide precursor POMC. POMC itself can be processed to produce a range of signals, some of which suppress food intake. POMC levels can be regulated by a variety of hormonal and nutrient signals that originate in the periphery. Glucose, for example, which would be high after a meal, acts to increase POMC transcription and suppress food intake. However, what has remained unclear is how nutrient signals such as glucose, amino acids, and fatty acids are converted into changes in the levels of neuropeptide transcripts.

## Unexpected Central Regulation of Metabolism by HIF

A role for HIF in the central control of metabolism was perhaps unexpected. The first suggestion that HIF may actually be acting centrally to control whole organism metabolism came from in vivo studies into the FIH knockout (KO) mouse. The FIH KO mouse exhibited a hypermetabolic phenotype, with greatly increased food intake, energy expenditure, and ventilation rate, in addition to reduced fat mass and improved insulin sensitivity. Surprisingly, a neuronal-specific FIH KO mouse recapitulated much of the phenotype of the total FIH KO mouse, suggesting a centrally mediated role for HIF signalling in the control of whole-organism energy balance [16]. However, while the phenotype of the neuronal-specific FIH KO mouse suggested a role for HIF within the brain, there were many alternative explanations for how FIH was acting. Firstly, FIH was deleted in all neurons, not just neurons in the central nervous system. Hence, FIH may have been acting in neurons of the carotid body, a key oxygen sensing node in the periphery. In addition, FIH does not exclusively regulate HIFα subunits having been shown to modify a range of proteins including Notch receptors, p105, IkBa, and SOCS4 [16].

In their elegant new study published in this issue of *PLoS Biology*, Zhang and colleagues [17] unequivocally demonstrate a role for HIF signalling in the hypothalamus and its ability to regulate peripheral energy balance. They demonstrate that HIF can directly activate the critical regulator of energy balance, POMC. Unlike other transcription factors previously shown to regulate hypothalamic neuropeptides, HIF regulates responses to nutrients such as glucose, as opposed to hormones such as leptin. Furthermore, ablation of HIF in POMC neurons of the arcuate nucleus of the hypothalamus resulted in a hyperphagic-hypometabolic animal with elevated fat mass. Conversely, overexpression of HIF in POMC neurons led to a hypermetabolic phenotype with resistance to high-fat diet–induced obesity.

## What Regulates HIF in the Brain under Normoxic Conditions?

Much of the focus into HIF function in the brain has come from investigations of its role in stroke. HIF is important for reducing intracellular metabolic rate in the face of the severe oxygen depletion that occurs during ischaemia. However, this paradigm makes little sense in the context of day to day metabolism, when oxygen levels in the brain only fluctuate to a small degree.

Considerable work has demonstrated that HIF stability and expression can be regulated by a wide variety of different signals, not necessarily related to oxygen levels. Two in particular, with relevance to the central control of metabolism, are glucose and insulin. Insulin is able to increase HIF1α protein levels [18], and glucose has been shown to regulate HIF through multiple mechanisms. First, glucose metabolites such as pyruvate can inhibit PHDs to stabilise HIFα [19]. Equally, as demonstrated by Zhang et al. [17] and others, TCA-cycle intermediates such as fumarate and succinate can promote HIF protein maturation by inhibiting PHDs. Furthermore, HIF has recently been shown to be upregulated by glucose through the carbohydrate response element binding protein (ChREBP) [20]. Conversely, prolonged hyperglycaemia has been suggested to destabilise formation of HIF dimers by methylglyoxalation of HIF1α [19]. It remains to be determined how many of these glucose-dependent effects on HIF occur within the hypothalamus, as well as their potential impact on energy balance.

## Signalling Pathways That Converge on HIF

In addition to showing that glucose-induced transcription of POMC was HIF dependent, Zhang et al. [17] also demonstrated that two key signalling pathways acted upstream of HIF to regulate this process. By blocking either the mTOR pathway or constitutively activating the AMPK pathway, Zhang et al. demonstrated that AMPK and mTOR are necessary for glucose-dependent upregulation of HIF2α. Furthermore, Zhang et al. go on to demonstrate that, in mice lacking HIF2α in POMC neurons, the effects of both the AMPK and mTOR pathways on food intake were blunted. In addition to glucose sensing, AMPK and mTOR have also been shown to be involved in fatty acid [21] and branched-chain amino acid–dependent effects [22] on whole organism energy balance. To what extent AMPK- and mTOR-dependent effects on energy balance are mediated through HIF activation remains to be determined.

## Outstanding Questions and Future Directions

**Figure 2:**
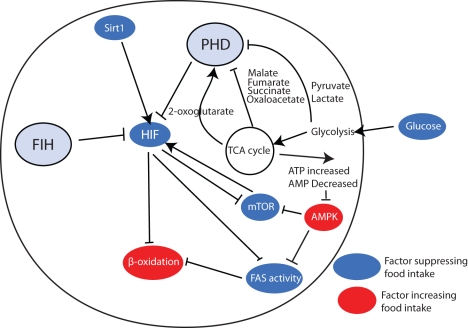
Potential links between HIF signalling and activity and known hypothalamic regulators of food intake. HIF can both be regulated by, and itself regulate, multiple pathways within the hypothalamus, which have been shown to regulate food intake. In general, activators of HIF tend to suppress food intake, in line with the anorectic effects of HIF put forward by Zhang et al. Two nodes of complexity are that HIF activation suppresses mTOR signalling, which would be expected to increase food intake. This may represent a negative feed-back loop. Second, HIF represses fatty acid synthesis (FAS), which has been shown to suppress food intake. However, inhibition of FAS acts through increasing β-oxidation, which is independently suppressed by HIF, potentially making this control point irrelevant.

It remains to be to determined if HIF plays a role in populations of neurons within the brain other than POMC neurons of the arcuate hypothalamus. So far, HIF has been demonstrated to mediate the response to glucose. Whether HIF is involved in the integration of the sensing of other nutrients and signals involved in the regulation of energy balance will be of considerable future interest (Figure 2).

A more fundamental question surrounding the role of HIF in the hypothalamus is whether HIF regulation represents a hypoxia-independent pathway or if glucose sensing is simply another part of an integrated metabolic response, common to both the normoxic and hypoxic roles of HIF. It is notable that genetic states of HIF activation in the hypothalamus—either direct overexpression [17] or neuronal FIH ablation [16]—lead to hypermetabolism. Hypermetabolism in the face of elevated HIF activity would seem to be a contradictory response, as under conditions of low oxygen one would expect an organism to reduce energy expenditure. However, it is important to note that these models have been phenotyped under normoxic conditions. It is possible that upregulation of metabolic rate, including hyperventilation and cardiac output, in these models is designed to provide oxygen to organs to maintain vital homeostatic processes such as heat production by brown adipose tissue. In the context of normoxia, the system is driven artificially quickly and energy expenditure runs at a much higher rate than it would in an actual hypoxic state. Alternatively, it could be that the multiple pathways that converge on and are regulated by HIF represent a convenient molecular machine to attach additional levels of regulation onto. If this is the case, then glucose and nutrient sensing may represent entirely hypoxia-independent regulatory mechanisms. While much future work will be needed to resolve these and other issues, it is clear that HIF proteins have an important role in the central control of metabolism.

## Abbreviations

FIH: factor inhibiting HIF
HIF: hypoxia-inducible factor
KO: knockout
PHD: proline hydroxylase domain
POMC: proopiomelanocortin
